# *Gynura segetum* induces hepatic sinusoidal obstruction syndrome in a child: A case report

**DOI:** 10.1097/MD.0000000000037341

**Published:** 2024-03-15

**Authors:** Qian Zheng, Haiyang Zhang

**Affiliations:** a Department of Pediatrics, West China Second University Hospital, Sichuan University, Chengdu, China; b Key Laboratory of Birth Defects and Related Diseases of Women and Children, Sichuan University, Ministry of Education, Chengdu, China.

**Keywords:** anticoagulant therapy, *Gynura segetum*, hepatic sinusoidal obstruction syndrome, hepatic veno-occlusive disease, pyrrolizidine alkaloids

## Abstract

**Rationale:**

Hepatic sinusoidal obstruction syndrome (HSOS), which includes hepatic stasis and portal hypertension, is a rare vascular disorder of the liver. It is often associated with hematopoietic stem cell transplantation. It is also possible to treat this disease using Chinese herbal medicines that contain pyrrolizidine alkaloids (PAs). This disease is extremely rare in children and poses a serious threat to their health. To our knowledge, this is the first case of HSOS in a child with PAs.

**Patient concerns:**

We report a 4-year-old boy suffering from abdominal pain, hepatomegaly, massive ascites, elevated liver enzyme level, and severe portal hypertension as a result of the consumption of Gynura segetum (also known as Tusanqi in Chinese, a traditional herbal medicine containing PAs).

**Diagnoses:**

The child was finally diagnosed with PA-HSOS based on pathological diagnosis and imaging examination.

**Intervention:**

With active symptomatic and supportive care and sequential anticoagulation therapy, the abdominal distension and liver function improved in the patient.

**Outcomes:**

The patient was eventually recovered. The levels of liver enzymes, hemoglobin, and bilirubin were normal, and the international normalized ratio fluctuated between 2.0 and 3.0 during 1-year follow-up after discharge.

**Lessons:**

This case report emphasizes the prevention of Chinese herb-induced liver injury in children and the importance of active long-term sequential anticoagulant therapy to reduce the progressive damage of PA-HSOS in the liver.

## 1. Introduction

Hepatic sinusoidal obstruction syndrome (HSOS), also known as hepatic veno-occlusive disease, causes liver injury, intrahepatic congestion, and post-sinusoidal portal hypertension.^[1–3]^ In HSOS, endothelial cells are edematous, necrotic, and exfoliated, with subsequent microthrombosis in the hepatic sinusoids, hepatic veins, and interlobular veins.^[4]^ It is characterized by abdominal distension, ascites, hepatomegaly, and hyperbilirubinemia.^[4–7]^ In certain instances, children with severe HSOS may progress to multi-organ dysfunction (MODS), ultimately resulting in death.^[8]^

The occurrence of HSOS after hematopoietic stem cell transplantation (HSCT) or chemotherapy with oxaliplatin in developed countries has been well documented.^[4,9–11]^ In children, the overall incidence of HSOS is 22% to 30%, notably higher than that in adults.^[8]^ However, the incidence reported in different studies can vary widely, ranging from 2% to 60%.^[8,12]^ It is alarming to note that 30 to 60% of children with HSOS may develop MODS within a few weeks after being affected by the condition.^[8]^ The mortality rate directly associated with HSOS is 15.5%, but this figure escalates to over 80% for cases in which HSOS advances to severe MODS.^[8,12]^ The most common cause of HSOS in China is the consumption of herbal or dietary supplements containing pyrrolizidine alkaloids (PAs).^[1,6,13]^ In 1920, Willmot reported the first case of HSOS caused by PAs. It was caused by Senecio tea, which contains PAs.^[14]^ It has been reported that the mortality rate of adults for PA-induced HSOS ranges from 16% to 40%, with liver failure being the common cause of death.^[15]^ However, the mortality rate among children remains unknown owing to the limited number of reported cases.

In this case report, we describe a child who presented with abdominal pain, abdominal distention, ascites, and severe portal hypertension and was eventually diagnosed with HSOS. This patient had consumed *Gynura segetum* (known in Chinese as *Tusanqi*, a PA-containing herbal remedy) previously. HSOS caused by PAs has not been reported in children.

## 2. Case presentation

The pediatric intensive care unit of West China Second Hospital received a 4-year-old boy on October 27, 2022. The patient presented with 18 days history of abdominal pain associated with progressive abdominal distention accompanied by persistent weakness, poor appetite, and intermittent vomiting. The patient had no fever, unconsciousness, jaundice, or bowel changes. The patient was orally intaking 100 mL of *G segetum* 3 days before the onset of the disease. The patient had no family medical history of abdominal tumors or hepatitis B infection. Since birth, the patient’s growth and developmental progress were normal.

**Figure 1.**
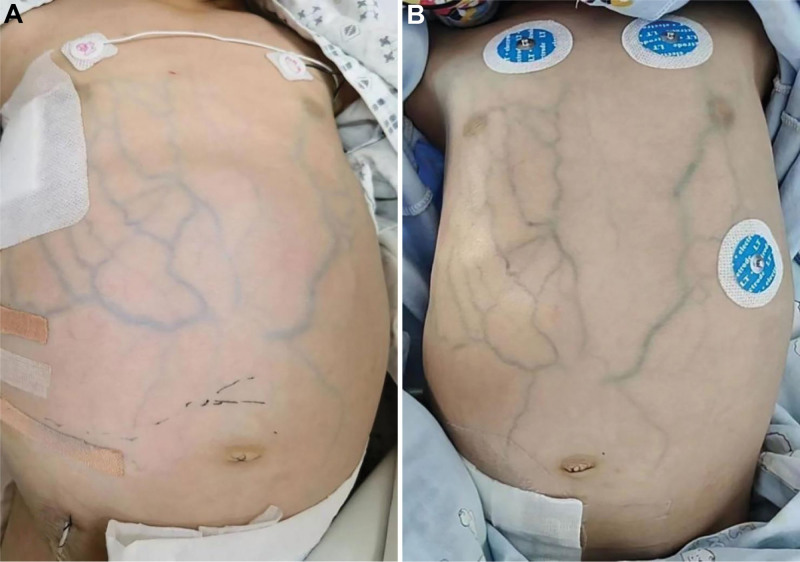
(A) Extreme abdominal distention and dilated superficial abdominal veins on admission (before ascites puncture). (B) Abdominal distension significantly relieved, but the dilated superficial abdominal veins were still visible (after ascites puncture).

Physical examination revealed a 3-concave sign, jugular venous distension, extreme abdominal distention (umbilical circumference and maximum abdominal circumference were 63 cm and 65 cm, respectively) with high tension, dilated superficial thoracic and abdominal veins (Fig. 1A), and hepatomegaly. Physical examination revealed a liver 8 cm below the right costal area and 12.5 cm below the xiphoid process, but no palpable spleen. The patient’s abdominal shifting dullness was positive, and bowel sounds were significantly reduced. The patient had no jaundice of the skin or sclera. The superficial lymph nodes were not palpably swollen.

**Figure 2.**
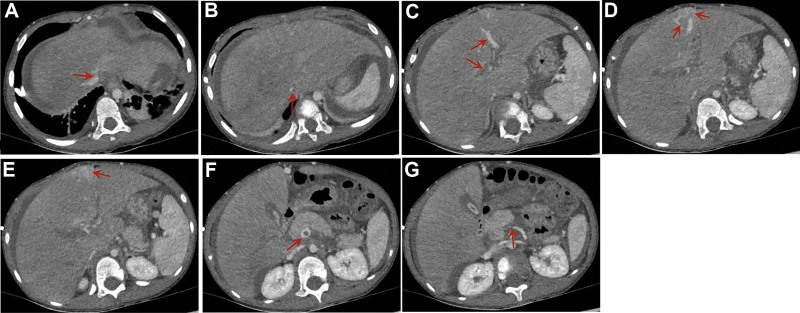
Computed tomography angiography. (A) Unclear display of hepatic veins (red arrow). (B) Hepatomegaly, massive ascites, heterogeneous enhancement of the liver, and stenosis of the hepatic segment of the inferior vena cava (red arrow). (C and D) The right branch of the portal vein was unclear, while the left branch was enlarged, anastomosing with the tortuous and thickened umbilical vein and abdominal wall vein (red arrow). (E) Patchy liver enhancement (red arrow). (F and G) The hypodense shadow of the portal vein and the superior mesenteric vein (red arrow).

**Figure 3.**
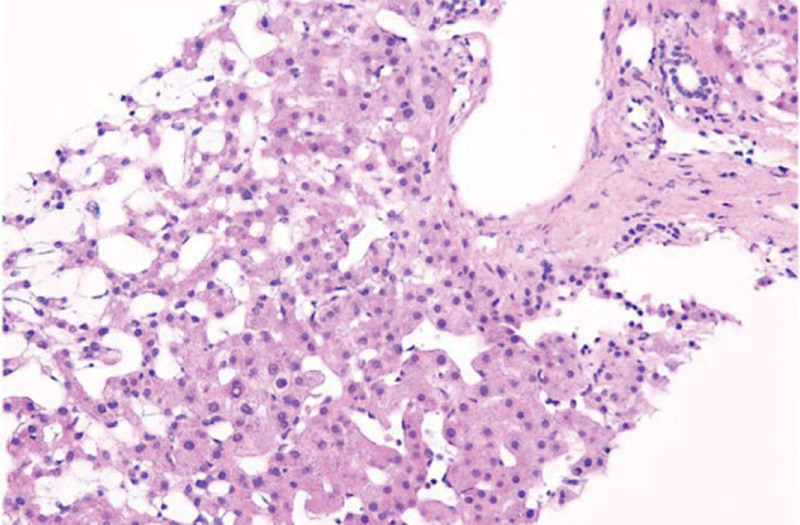
Liver biopsy: dilation of hepatic sinusoidals, hydropic degeneration of hepatic cells, potty necrosis scattering in the hepatic lobule sinusoid, and a few inflammatory cell infiltrations in the portal area (Hematoxylin-eosin stains, ×200).

Laboratory tests revealed deteriorated liver function: total bilirubin was 19.6 μmol/L (reference 5–23 μmol/L), direct bilirubin was 9.3 μmol/L (reference: <8 μmol/L), indirect bilirubin was 10.4 μmol/L (reference <17 μmol/L), alanine aminotransferase was 1278 U/L, aspartate transaminase was 1746 U/L, total protein was 35 g/L (reference 62–76 μmol/L), serum albumin was 28.5 g/L (reference 38.0–54.0 μmol/L), lactic dehydrogenase (LDH) was 1106 U/L, blood ammonia was 97.3 μmol/L, γ-transpeptidase was 58 U/L, and bile acid level was 184.8 μmol/L. Coagulation function deteriorated significantly. The prothrombin time was 19.3 seconds, activated partial thromboplastin time was 44.2 seconds, international normalized ratio was 1.43, D-dimer was 20.03 μg/mL, fibrinogen was 76 mg/dL, and fibrinogen degradation products was 55.42 μg/mL. Routine blood examination revealed a high White blood cell count of 14.35 × 10^9^/L and high C-reactive protein of 45.72 mg/L, but a low platelet count of 20 × 10^9^/L and a low hemoglobin level of 88 g/L. Puncture examination of the ascites revealed that the fluid was transudate, and we performed a bedside ultrasound-guided abdominal puncture after subcutaneous anesthesia with 0.5% lidocaine. A total of 800 mL of yellow, non-cloudy ascites was aspirated via abdominal puncture. Abdominal distension was significantly relieved after abdominal puncture, but dilated superficial abdominal veins were still visible (Fig. 1B). The laboratory testing of ascites revealed the following results: the total nucleated cell count was 51 × 10^6^/L, the ascites albumin was 4.9 g/L, the serum ascites albumin gradient (SAAG) was above 11 g/L, LDH was 100 U/L, adenosine deaminase was 0.7 U/L, and the ascites bacterial culture was negative. The abdominal ultrasound report shows: the liver was enlarged, with the right lobe’s maximum oblique diameter measuring approximately 13.6 cm; fatty liver is observed, along with hepatic parenchymal damage, and no tumor-like enhancement is detected; the gallbladder wall is edematous and thickened; the pancreas appears slightly full, with increased parenchymal echoes and slightly indistinct margins; no significant abnormalities are seen in the spleen or both kidneys; a large amount of ascites is present in the abdominal cavity, with a depth of approximately 4.8 cm. Abdominal computed tomography (CT) revealed hepatomegaly, hydrothorax, and ascites (depth was 6–8 cm). Abdominal computed tomography angiography (CTA) revealed abnormal signs of portal hypertension (Fig. 2). We further performed hepatic vein and inferior vena cava cavography, which suggested that the blood vessels were unobstructed, but the bilateral hepatic veins were narrow. A liver biopsy was performed, and histopathological analysis revealed hydropic degeneration of hepatic cells, potty necrosis scattered in the hepatic lobule, prominent extension of the partial hepatic sinusoid, and a few inflammatory cell infiltrations in the portal area (Fig. 3).

**Figure 4.**
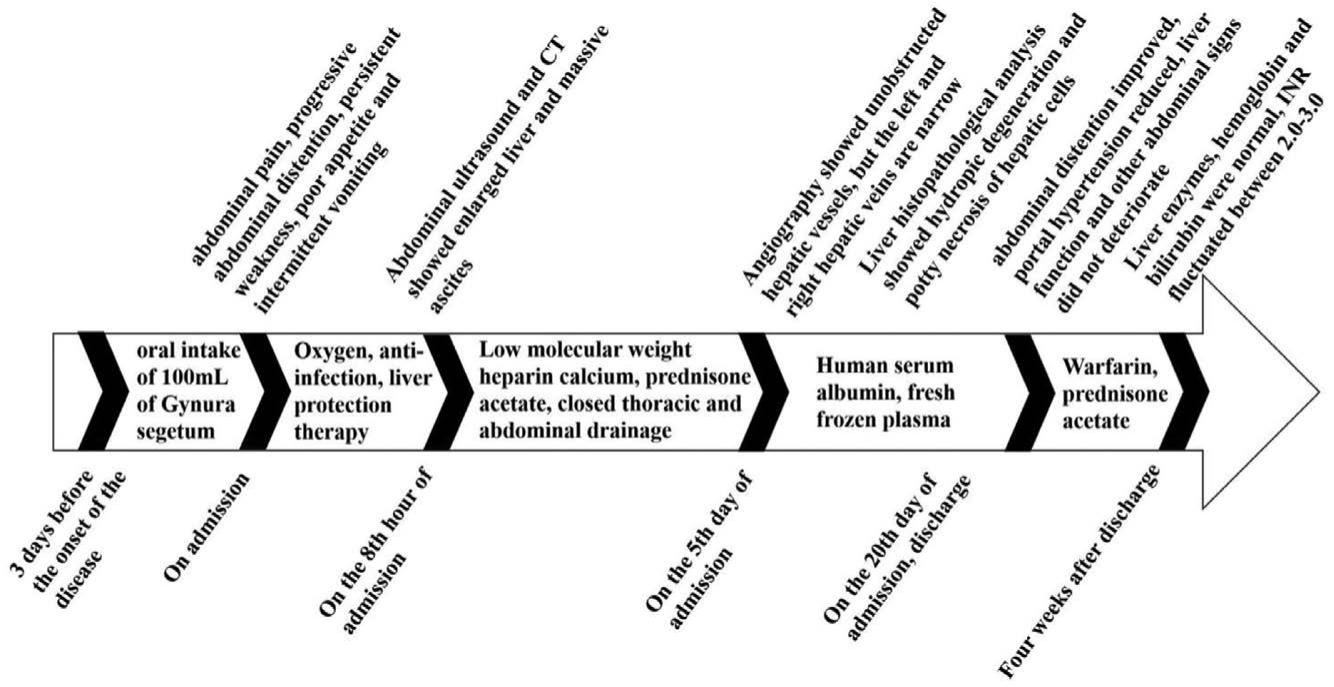
The timeline of the treatment and disease progression.

Based on the patient’s history of Chinese herbal medicine intake, main symptoms, and laboratory and imaging examinations, HSOS was finally diagnosed. After admission, he received active symptomatic and supportive treatment, including reduced glutathione for liver protection and prednisone acetate (1 mg/kg/day for 20 days) for oral anti-inflammatory effects. Albumin infusion (10 mg qd) and fresh frozen plasma (20 mL/kg/d qd) were administered for 3 days. Anticoagulation was administered by subcutaneous injection of low molecular weight heparin calcium (100 IU/kg/d q12h for 20 days). Continuous peritoneal puncture drainage was not performed because of the poor coagulation function. During conservative treatment, we discussed with the liver surgeon on several occasions that the child did not meet the indications for transjugular intrahepatic portosystemic shunt or liver transplantation because irreversible liver failure had not developed. Through the above active anticoagulation and low-dose hormone therapy, abdominal distension and ascites were relieved after 20 days. Meanwhile, the liver was reduced to 5 cm subcostal and 6 cm subxiphoid. Liver enzyme, serum albumin, and blood ammonia levels returned to normal levels. Superficial abdominal veins were relieved. Reexamination with CTA indicated that the liver was significantly reduced, ascites disappeared, and the low-density shadow of the hepatic portal vein was significantly reduced. The patient was discharged from the hospital and continued to take warfarin (0.1–0.2 mg/kg/d qd) and prednisone (1 mg/kg/d for 7 days and then 0.5 mg/kg/d for 7 days) orally at home. After discharge, outpatient follow-up were conducted every 2 weeks for a year. The levels of liver enzymes, hemoglobin, and bilirubin were normal, and the international normalized ratio fluctuated between 2.0 and 3.0. The timeline of treatment and disease progression is summarized in Figure 4.

## 3. Discussion

HSOS is an unusual hepatic disease with post-sinusoidal portal hypertension induced by injury to the hepatic sinusoidal endothelium and obstruction of the hepatic sinus outflow tract.^[7]^ In Europe and the United States, HSOS has been widely considered an adverse reaction caused by cytoreductive therapy before HSCT,^[16,17]^ chemotherapy for solid tumors,^[18–20]^ and immunomodulator agents after orthotopic liver transplantation.^[21]^ In China, the oral administration of PA-containing plants, for example, *G segetum*, was the main cause of HSOS.^[1,4,6,13]^ The patient in this case report did not receive HSCT, chemotherapy, or immunosuppressive agents. Only oral intake of *G segetum* appears to be related to this disease. This is the first pediatric patient reported to have been diagnosed with HSOS after receiving *G segetum*.

*G segetum* is a traditional Chinese herbal medicine with hemostatic and analgesic functions that has great value in medicine and health care. Many alkaloids have been isolated from *G segetum* and PAs are known to be the most important cause of HSOS.^[22]^ Dehydropyrrolizidine alkaloids and dehydroretronecine are the main metabolites of PA with obvious hepatotoxicity.^[4,22]^ Associating them with proteins within cells and nucleic acids can lead to cell death. Hepatic sinusoidal endothelial cells, especially in zone III of the liver acinus, are the main targets of PA metabolites.^[22]^ The swelling, damage, and shedding of hepatic sinusoidal endothelial cells attacked by these poisonous metabolites can block sinusoidal flow. As blocking and stagnation intensified, post-sinusoidal portal hypertension occurred.^[4,22,23]^

The most prominent symptoms of PA-HSOS are jaundice, fatigue, anorexia, abdominal bulge, bellyache, hepatomegaly, and ascites.^[4,23,24]^ However, symptoms of PA-HSOS in children are often atypical. In this case, the patient only had atypical clinical presentations such as abdominal pain and abdominal distension without jaundice. In most patients, the routine blood test results are generally normal. However, with infection, elevated White blood cell counts and anemia can be seen.^[23]^ Patients with PA-HSOS are at a risk of developing severe thrombocytopenia.^[23]^ Abnormal liver function, including hypoalbuminemia and increased serum bilirubin levels.^[4,23]^ The coagulation function of PA-HSOS often results in an elevated D-dimer level, mild extension of prothrombin time, and activated partial thromboplastin time.^[23,24]^ In this case, there was an elevation in White blood cells, C-reactive protein, liver enzymes, LDH, blood ammonia, bile acid, and D-dimer levels, accompanied by anemia, thrombocytopenia, and hypoalbuminemia. These auxiliary examination results were consistent with the typical presentation of HSOS. Ascites tests in patients with HSOS usually present a SAAG of >11 g/L, indicating portal hypertensive ascites.^[23,24]^ The SAAG for this child was 23.6 g/L, and the total ascitic cell count was <100 × 10^6^/L, predominantly consisting of mononuclear cells, which is consistent with the characteristics of exudative ascites. Abdominal imaging plays an important role in the diagnosis of HSOS. Zhou et al conducted a study of 16 consecutive *G segetum*-induced HSOS cases in 2014 and concluded that ascites, hepatomegaly, patchy liver enhancement, main hepatic vein narrowing, and thickening of the gallbladder wall were significant CT features of PA-HSOS.^[25]^ Abdominal imaging should adopt a diversified approach rather than just referring to a single result, such as abdominal ultrasound or CT. In this case, abnormal signs of portal hypertension were first detected on CTA, and the blood vessels were unobstructed on endovascular angiography. Besides, clinical severity was positively associated with the abnormal patchy liver enhancement grade, according to their study.^[25]^ Recently, the above findings were confirmed in large sample sizes by Kan et al and Zhuge et al.^[4,23,26]^

The current PA-HSOS diagnostic criteria are the *Nanjing criteria*.^[27]^ First, the patient must have a confirmed history of PA-containing herb intake, and the diagnosis should contain pathological evidence or three of the following 3 diagnostic elements: (1) abdominal bulge, hepatomegaly, pain in the hepatic region, and ascites; (2) increased serum bilirubin levels or other deteriorating liver function; (3) enhanced CT or magnetic resonance imaging features; and (4) pathological examination of the rigid viscera_._^[23]^ In this case, the patient satisfied the diagnostic criteria. Younger children are often unable to state whether they have a history of taking *G segetum* and other PA-containing herbal medicines, so we should inquire with their guardians about their medication history. It was only after further questioning that we became aware of the patient’s history of taking *G segetum*. Once PA-HSOS is suspected in patients with a vague medication history, toxicology testing can be performed to help diagnose the condition. Moreover, it is necessary to eliminate additional potential etiological factors for liver damage before making a diagnosis. In the diagnostic process of this case, we first ruled out common etiologies of liver dysfunction, such as infections, parasites, and tumors. The differential diagnosis mainly includes Budd-Chiari syndrome (BCs) in conjunction with the patient’s medication history. They are both characterized by post-sinusoidal portal hypertension, but have different etiologies and clinical features. HSOS often occurs after PA-containing herb intake in China or HSCT, whereas patients with BCs often have a medical history of chronic liver fibrosis with unknown causes.^[23]^ The main symptom of HSOS is acute liver damage, whereas BCs are often found at the stage of decompensated liver cirrhosis with splenomegaly and hypersplenism.^[24]^ The CT images of HSOS are mainly classified according to ascites, hepatomegaly, and typical map-like heterogeneous enhancements.^[25]^ Moreover, liver enlargement constricts the vital hepatic veins or inferior vena cava and causes stenosis, but the blood flow is unobstructed, and communicating branches between the narrowed hepatic veins are not observed. In contrast, the CT of BCs mainly manifests as splenomegaly and liver cirrhosis, significant occlusion or stenosis of the hepatic veins, and inferior vena cava with open collateral circulation. In terms of pathology, HSOS is characterized by cell swelling and shedding, whereas BCs mainly lead to peripheral sinus and interlobular vein sclerosis and collagen deposition, but not endothelial cell degeneration.^[23,24]^ Ultimately, based on the medication history, symptoms of acute liver injury, and characteristic radiological and pathological changes, the patient was diagnosed with HSOS rather than BCs.

The current treatments for PA-HSOS are limited. In general, the suggested therapeutic principles include exposure avoidance, symptomatic treatment, anticoagulant therapy, surgical operations, and liver transplantation (Table 1).^[4,22,23,28–31]^ Fortunately, PA-HSOS is often irreversible in children because of the low intake of PA-containing herbs. Intrahepatic congestion, damage to hepatic function, and portal hypertension can be effectively alleviated by timely diagnosis and adequate anticoagulant treatment. In this case, the patient received early and sequential anticoagulant therapy. Finally, his abdominal pain disappeared, abdominal distention greatly improved, portal hypertension was reduced, and liver function and other abdominal signs did not progressively deteriorate before discharge. Because of his good reaction to medical therapy, the surgeon considered that surgery was not necessary for the time being.

**Table 1:** Suggested therapeutic principles for PA-HSOS.

Suggested	Comments
Exposure avoidance	Termination of PA-containing herbs exposure is the first step.^[4,23]^
Symptomatic treatment	Supportive symptomatic treatment is the essential but especially important scheme, including liver protection, diuretic therapy, albumin infusion, drainage of ascites, improvement of microcirculation, and so on.^[4,23]^
Anticoagulant therapy	Anticoagulant therapy is recommended in guidelines to be applied to acute or subacute HSOS patients unless contraindicated.^[4,23]^ Low molecular weight heparin is the first medication regimen. Warfarin may be used concurrently with low molecular weight heparin or orally sequentially after low molecular weight heparin is discontinued.^[4,24]^ In addition, many recent studies at home and abroad have confirmed anticoagulant therapy’s effectiveness.^[23,28,29]^
Surgical operation	TIPS is confirmed to present a safe and effective treatment for patients with poor feedback on the efficacy of the above treatment options and can improve portal hypertension and ascites.^[23,29]^ However, there remain controversies about whether TIPS can improve the long-term prognosis.^[22,30,31]^
Liver transplantation	Liver transplantation is currently the best treatment option for many end-stage liver diseases. In the guidelines, it is also recommended in severe cases of PA-HSOS with liver failure.^[23]^

HSOS = hepatic sinusoidal obstruction syndrome, PA = pyrrolizidine alkaloids, TIPS = transjugular intrahepatic portosystemic shunt

As the current diagnosis and treatment for this particular case demonstrated positive results, there is still room for improvement and a clear need for further investigation. A few limitations must be acknowledged in this report. This case report encompasses only a single pediatric patient, thus constraining the generalizability of the findings to a wider population. For more comprehensive future research, it would be beneficial to collect and analyze data from additional pediatric cases across various regions and conduct longitudinal studies to monitor the progression and enduring effects of PA-HSOS. Such efforts could foster a more nuanced understanding of these conditions. Furthermore, randomized controlled trials should be conducted to determine the most effective treatment options for PA-HSOS.

## 4. Conclusion

*G segetum* is a traditional Chinese herb used to treat hepatotoxic PAs. Oral administration of this drug to children can cause PA-HSOS and seriously worsen liver function. This critical illness can be diagnosed early using pathology combined with imaging. Active long-term sequential anticoagulant therapy can reduce the progressive damage to PA-HSOS in the liver. In this case, we also call for avoiding the threat of PA-HSOS to children’s health at the source by eliminating the use of *G segetum* and other PA-containing herbal medicines as much as possible.

## Acknowledgments

The authors thank all the medical staff involved in the treatment of this patient and the implementation of this study.

## Author contributions

**Conceptualization:** Haiyang Zhang.

**Data curation:** Qian Zheng.

**Formal analysis:** Qian Zheng.

**Methodology:** Qian Zheng.

**Project administration:** Haiyang Zhang.

**Resources:** Haiyang Zhang.

**Software:** Qian Zheng.

**Validation:** Haiyang Zhang.

**Visualization:** Haiyang Zhang.

**Writing – original draft:** Qian Zheng.

**Writing – review & editing:** Haiyang Zhang.
